# Crystallographic–Morphological Connections in Star Shaped Metal–Organic Frameworks

**DOI:** 10.1021/jacs.2c09785

**Published:** 2022-12-12

**Authors:** Maria Chiara di Gregorio, Vivek Singh, Linda J. W. Shimon, Michal Lahav, Milko E. van der Boom

**Affiliations:** † Department of Molecular Chemistry and Materials Science, Weizmann Institute of Science, Rehovot 7610001, Israel; ‡ Department of Chemical Research Support, Weizmann Institute of Science, Rehovot 7610001, Israel

## Abstract

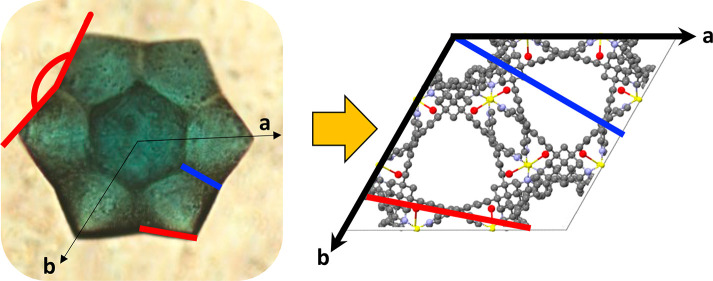

The symmetry of a crystal’s morphology usually reflects the symmetry of the crystallographic packing. For single crystals, the space and point groups allow only a limited number of mathematical descriptions of the morphology (forms), all of which are convex polyhedrons. In contrast, concave polyhedrons are a hallmark of twinning and polycrystallinity and are typically inconsistent with single crystallinity. Here we report a new type of structure: a concave polyhedron shape single crystal having a multidomain appearance and a rare space group (*P*622). Despite these unusual structural features, the hexagonal symmetry is revealed at the morphological levels.

The basic principles of crystallography were deduced from the analysis of crystal morphology.^1^ All crystal forms can be described by 32 different combinations of symmetry elements.^2^ These sets of symmetry are identical to the 32 point groups that describe the atomic symmetries.^3^ The correlation between the chirality of crystal morphologies and their crystallographic structures has been demonstrated by Pasteur,^4^ Donnay and Harker,^5^ and the more recent works of Lahav and Leiserowitz.^6,7^ The geometric symmetry of the external crystal habit is often related to its internal molecular symmetry.^8−10^ In other words, the point group symmetry of the crystal morphology is dictated by the crystal structure, i.e., the space group of the crystal.^11^

The description of crystals includes space groups (a set of symmetry elements related to the atomic disposition in the unit cell), point groups (a set of symmetry elements that operate on an infinite three-dimensional lattice so as to leave one point unmoved), and their related forms (a set of identical crystal facets related by the symmetry of the point group). These concepts allow one to correlate symmetries from the atom disposition to morphology and *vice versa*. Point groups are obtained by removing the translational elements from the space groups, and for each point group, only defined forms can exist. The crystallographic rules describe the symmetry of almost the entire range of known (bio)minerals and synthetic crystals.^12^ There are only a few exceptions; quasi-crystals have forbidden symmetries at the molecular level that are inconsistent with all space groups.^13^ Single crystals with a multidomain appearance are another intriguing class, having a complex, rather than polyhedral, habit. These crystals have external morphologies that defy the conventional crystal forms and have been observed in biomineralization.^14−16^ Other examples were obtained by using calcium carbonate and templating agents.^17−19^ Organic compounds can also exhibit this phenomenon, as shown by the seminal work of Kahr.^20^ Recently, we demonstrated this phenomenon by using metal–organic frameworks (MOFs) to form single crystals with curved and multidomain morphologies.^21,22^ The mechanisms involved in the formation of MOFs are highly complex.^23−26^

Here we report an unexpected crystallographic phenomenon: single crystals with a multidomain polyhedral morphology and concave (re-entrant) angles. According to crystallographic conventions, it is thought to be impossible to generate single crystals featuring re-entrant angles with any of the unit cell geometries.^27^ We show here that such a morphology with single crystallinity exists with a hexagonal unit cell in the rare space group *P*622.

The MOFs (**CSTAR-NO_3_** and **CSTAR-SO_4_**) were grown using solvothermal conditions in sonicated solvents (DMF/CHCl_3_, 3:1 v/v).^21,22^ Cu(NO_3_)_2_ or CuSO_4_ was reacted with the organic ligand, **AdDB**, in a molar ratio of 2:1 at 105 °C for 2 days. Additives and modulators are often used to influence and control the size and shape of crystals.^28,29^ However, here no additives were used to control the growth and the shape of the facets (Scheme 1A). Under the applied reaction conditions, the solvents are a source of radical species that can reduce the concentration of the active metal salt thereby influencing the crystallization process.^22^

**Scheme 1:**
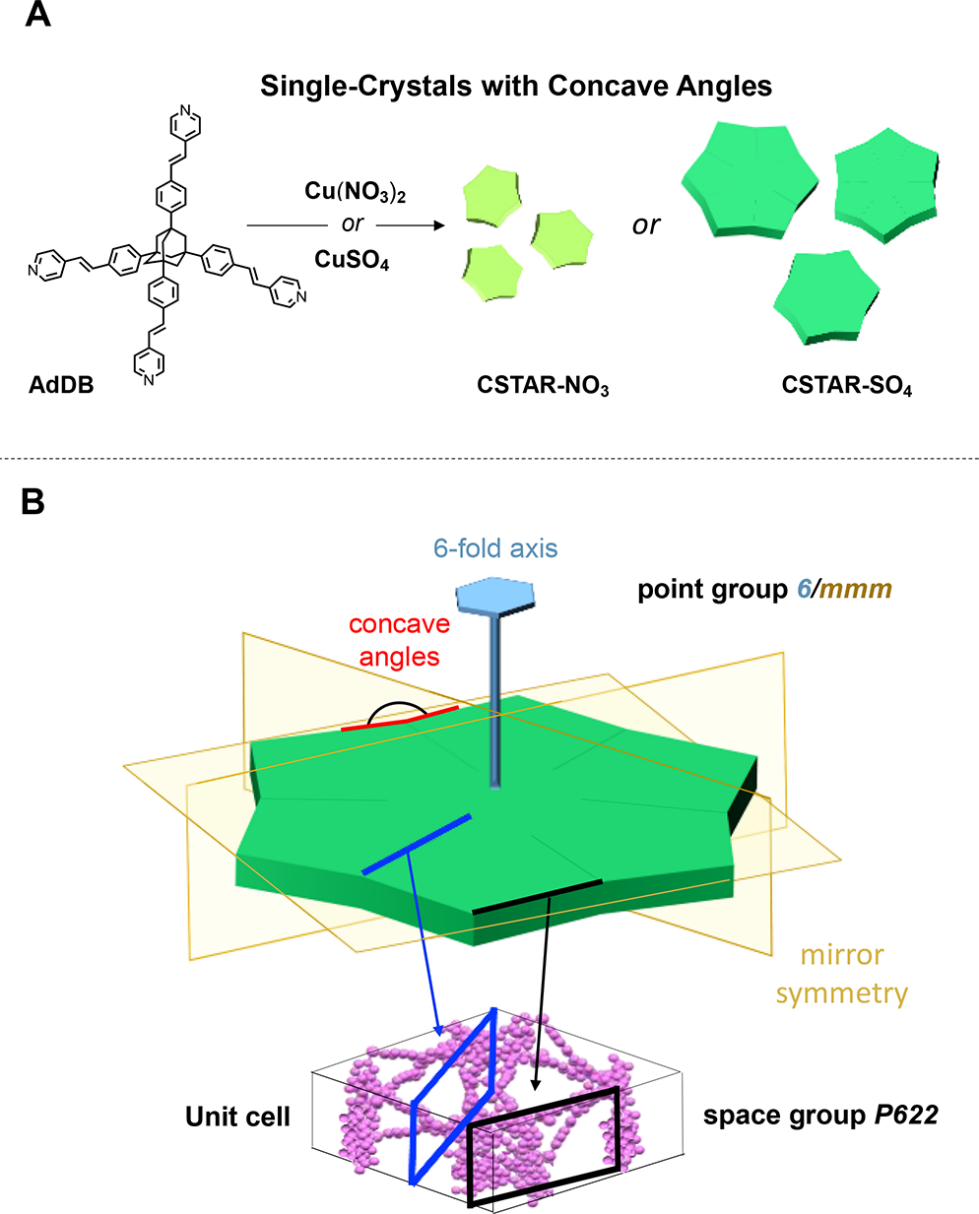
Concave and Multidomain Morphology Combined with Single Crystallinity: (A) Uniform and Star-Shaped Metal–Organic Frameworks Formed via Solvothermal Synthesis; (B) Symmetry Relationship between the Multidomain Morphology (Point Group 6/*mmm*) and the Molecular Structure (Space Group *P*622) Linking the Concave Facets and the Boundaries of the Morphological Domains to the Molecular Pattern of the Unit Cell

Optical and scanning electron microscopy (SEM) show the formation of a “stellate” prism (Figures 1, 2, S1–3). Regardless of the counterions (NO_3_^–^, SO_4_^–^), the crystals have a similar, uniform morphology and are monodispersed. Their concave polygon shape resembles a six-pointed star. Lateral views show that the center is domed. Such optically observable features indicate differences in extinction coefficients representing different crystal phases or orientation of the same phase.^30^ However, in this case these features are most likely to be the consequences of thickness and curvature changes in the crystals. The diameters of the **CSTAR-NO_3_** and **CSTAR-SO_4_**, measured as the distance between two opposite points, are 32 ± 6 μm and 177 ± 41 μm, respectively.

**Figure 1:**
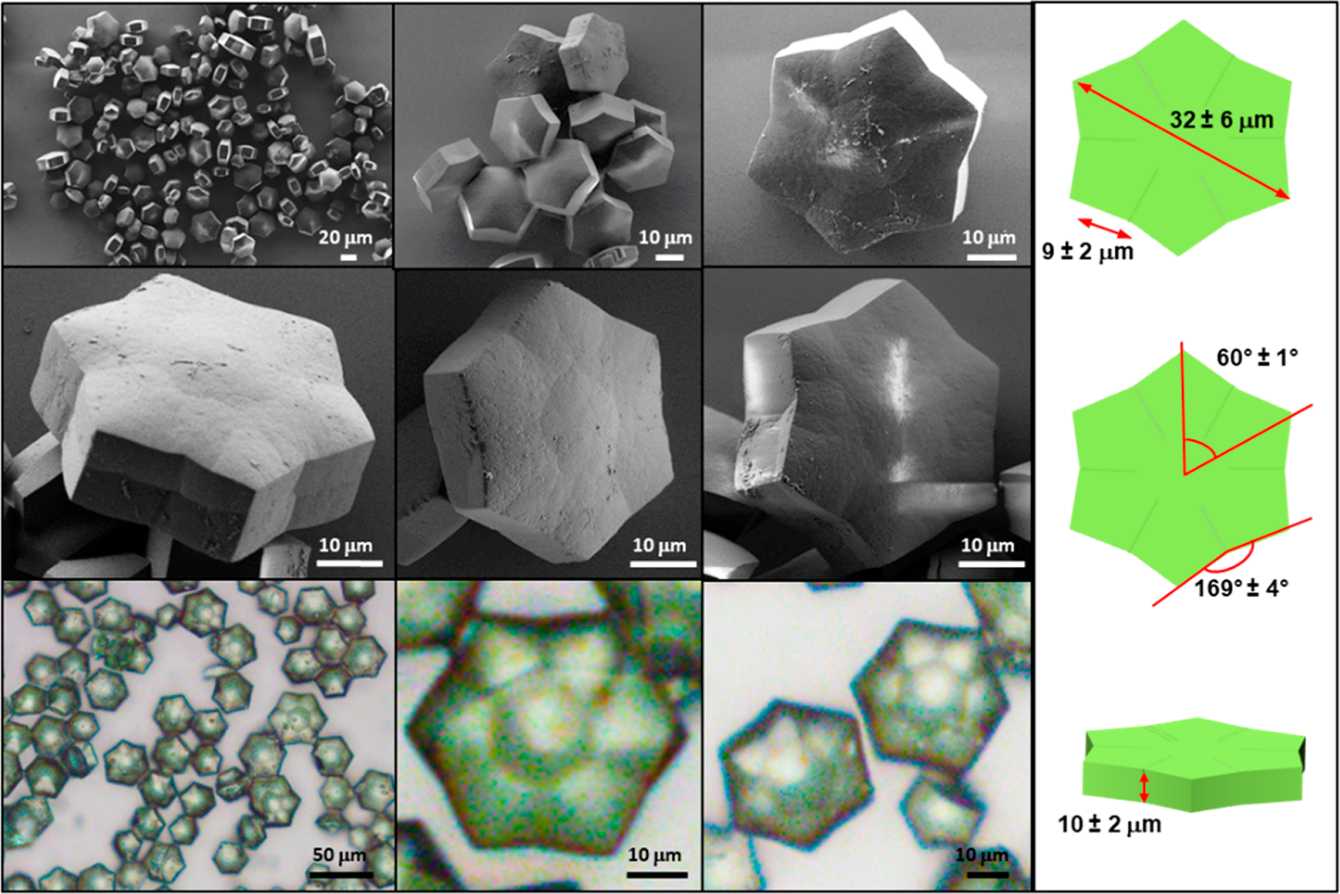
Scanning electron microscopy (SEM) and light microscopy images of **CSTAR-NO_3_**. For polarized images, see Figure S3.

**Figure 2:**
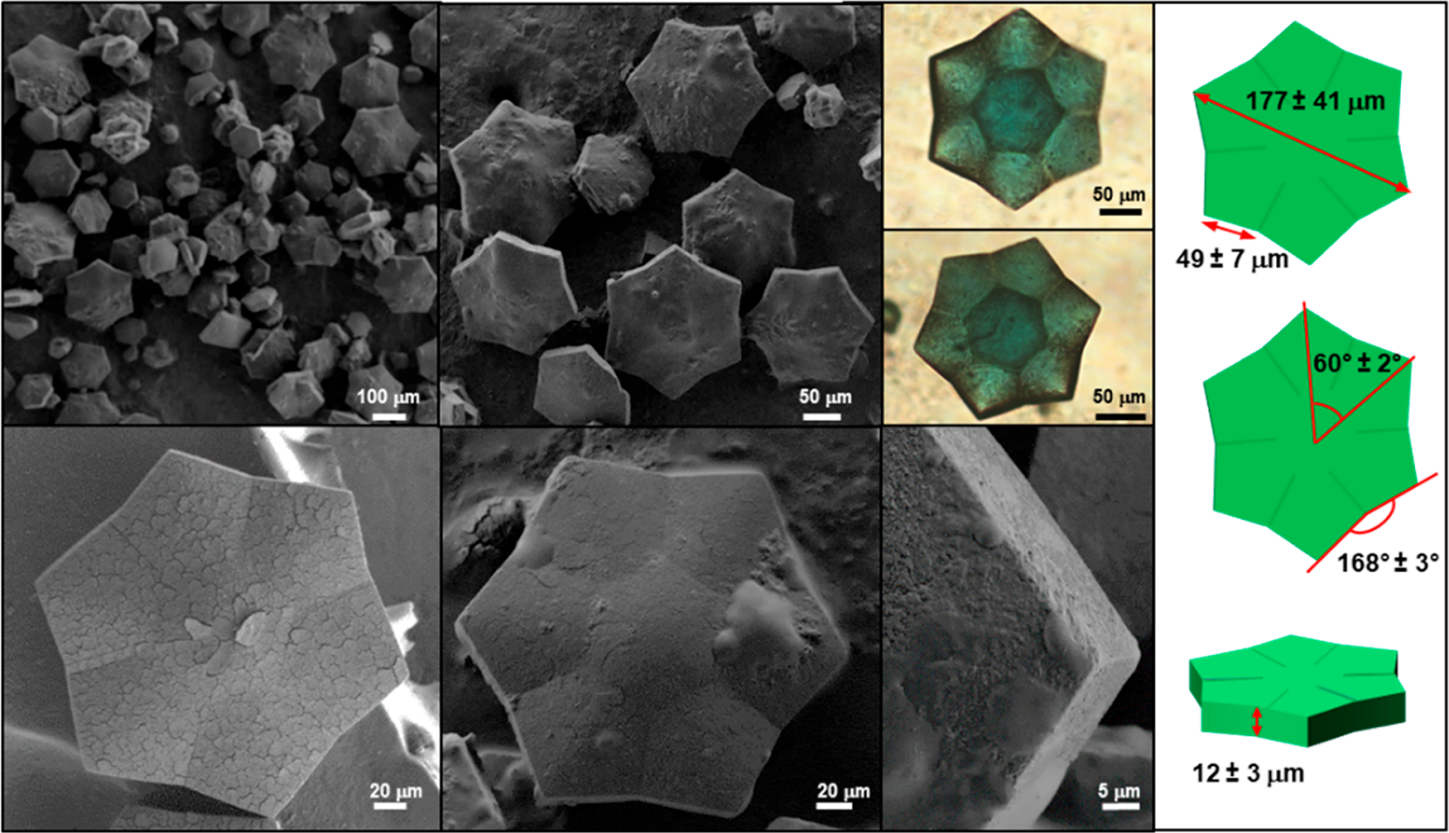
Scanning electron microscopy (SEM) and light microscopy images of **CSTAR-SO_4_**. For polarized images, see Figure S3.

The angles defined by adjacent points and the center of the **CSTAR-NO_3_** and **CSTAR-SO_4_** crystals are 60° ± 1° and 60° ± 2°, respectively, for at least 45 crystals. The length of the 12 facets are 9.4 ± 2.3 μm and 48.4 ± 8.6 μm for **CSTAR-NO_3_** and **CSTAR-SO_4_**, respectively, Figure S4). The morphology allows one to relate the crystal facets by symmetry elements of the 6/*mmm* point group, i.e., a 6-fold symmetry perpendicular to the stellate base, mirror planes parallel to the stellate bases and passing through the base vertices (Scheme 1B).

Based on crystallographic classifications and examples found in nature, the concave and multidomain appearance of our crystals would indicate a twinned and polycrystalline system.^31−35^ In contrast, we found that single crystals are formed. This claim is based on the analyses of the diffraction patterns of six whole crystals, including (i) the shape and intensity distribution of the diffractions, (ii) Flack parameters, and (iii) symmetry analysis (Laue statistics). These analyses were conducted using a single crystal X-ray diffractometer with a relatively large cross-section beam, 100 μm, ensuring that all sectors of the crystals are exposed throughout the diffraction experiment. Moreover, a single point of a star was cut, and the single crystal analysis gave similar results (**CSTAR-SO_4_**; Table S2, CCDC 2214170).

X-ray diffraction data from twinned crystals display well-established pathologies that can be used to identify twinning.^36,37^ Our diffraction data (Ewald’s sphere) show distinct spots and single defined patterns, fully compatible with a single-domain orientation (Figures 3A and S5). The crystal structures were solved to atomic resolution and fully refined (Tables S1, S2). The Flack parameters were close to zero, therefore excluding also the possibility of inversion twinning. Both enantiomeric forms of *P*622 were seen in the samples studied. The space group *P*622 is incompatible with the close packing of organic molecules and has a low occurrence in the CCDC database.^38−46^ The concave angles in a morphology normally associated with twinning motivated us to assess the intensity distribution and symmetry of the diffraction in order to exclude the possible presence of merohedral twinning phenomena.^47^ In the case of merohedral twinning, the reflections of the domains overlap exactly and, as a consequence, the diffraction pattern mimics single crystallinity. The diffraction data of merohedral crystals should exhibit (i) a deviation from the expected intensity distribution^37,48,49^ and (ii) a symmetry higher than that of the true Laue symmetry.^50^ Merohedral twinning was excluded using the following tests: (i) The average intensity distribution values, ⟨|*E*^2^ – 1|⟩, obtained from the intensities of the diffractions (∼0.8), indicated the formation of non-centrosymmetric structures, in line with the crystal structure determinations. For twinned crystals, a much lower value is expected (∼0.2). Furthermore, the cumulative intensity distribution plots are also in good agreement with non-centrosymmetric, single crystals (Figures 3B and S5). (ii) The symmetry of the crystals was examined. Due to the exact superpositioning of diffraction patterns, merohedral twinning simulates a higher symmetry, which leads to an apparently higher, but incorrect, Laue class. Merohedral twinning occurs in hexagonal and other crystal systems that have more than one Laue class.^51^ Our structural determinations indicated a hexagonal system with the point group 6/*mmm* and the space group *P*622. The point group of these crystals is expressed in the higher symmetry Laue class 6/*mmm*. A comparison of the calculated *R*_int_ values of the possible Laue groups showed that the assigned symmetry and space group are correct (Figure 3C) and not due to merohedral twinning.

**Figure 3:**
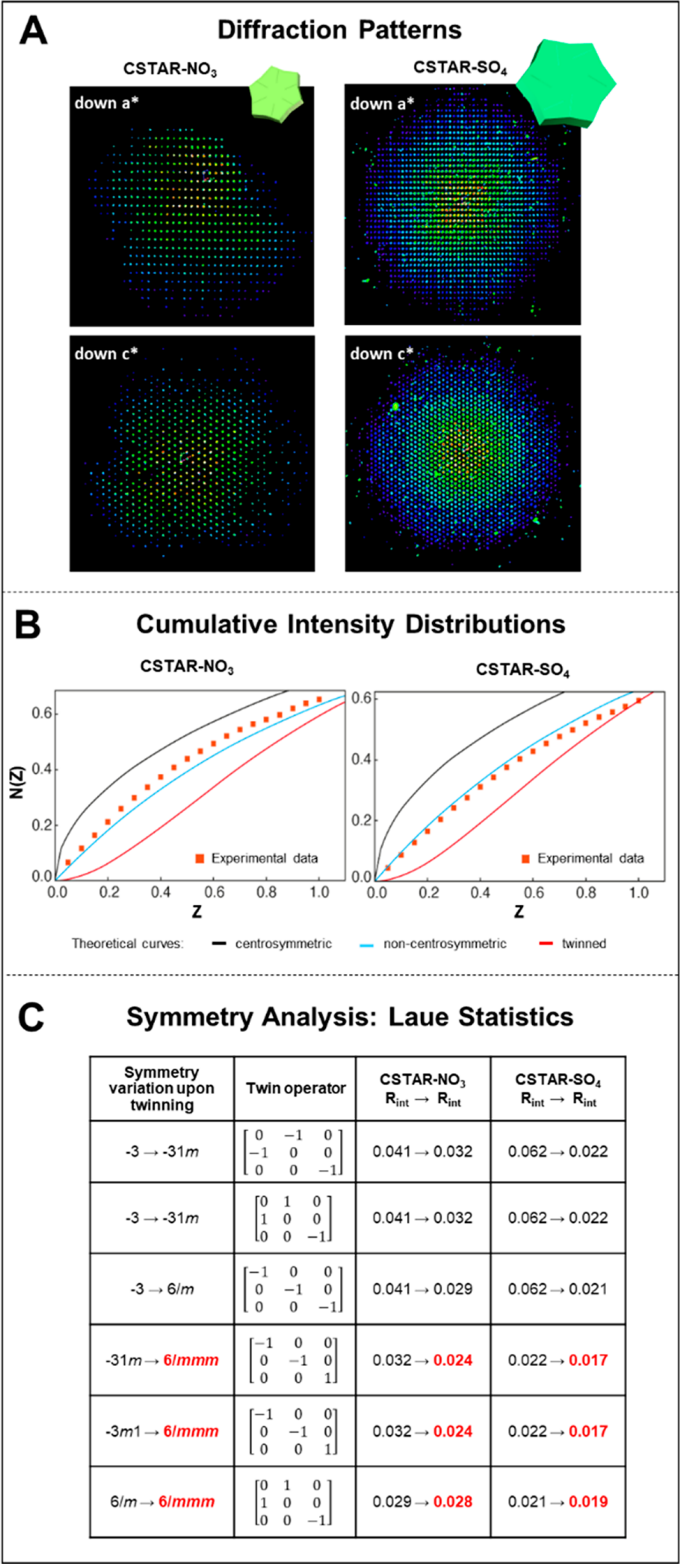
Multiple crystallographic tests demonstrating single crystallinity with exclusion of hidden twinning phenomena: (A) Ewald’s sphere projections, (B) cumulative intensity, (C) Laue class statistics of **CSTAR-NO_3_** (CCDC 2009649) and **CSTAR-SO_4_** (CCDC 2117030).

The single crystal X-ray structures of the seven samples show that the stellate crystals are chiral MOFs formed by a single continuous network (Figures 4, S6, and S7). The chirality is expressed at different levels: At the molecular level, the metal–pyridine coordination chemistry resulted in two types of helicoidal channels and homochiral metal nodes. Four pyridine units belonging to different ligands are arranged in a propeller-like fashion around the Cu^2+^ centers. The axial positions are occupied by coligands (water molecules, Cl, and/or SO_4_^2–^). The two sets of channels are parallel to the *c*-axis. Hexagonal channels centered around the 6-fold axis are surrounded by six identical trigonal channels. The shapes of the channels follow the 6-fold and 3-fold symmetry elements of the space group. Both enantiomers were observed having *P* or *M* helicity (Tables S1 and S2). No π–π interactions were found. The phase purity of the bulk was demonstrated by powder X-ray diffraction (PXRD) measurements (Figure S8). Thermogravimetric (TGA) analysis showed that both **CSTAR-NO_3_** and **CSTAR-SO_4_** have a similar high thermal stability and loss of solvents (Figure S9).

**Figure 4:**
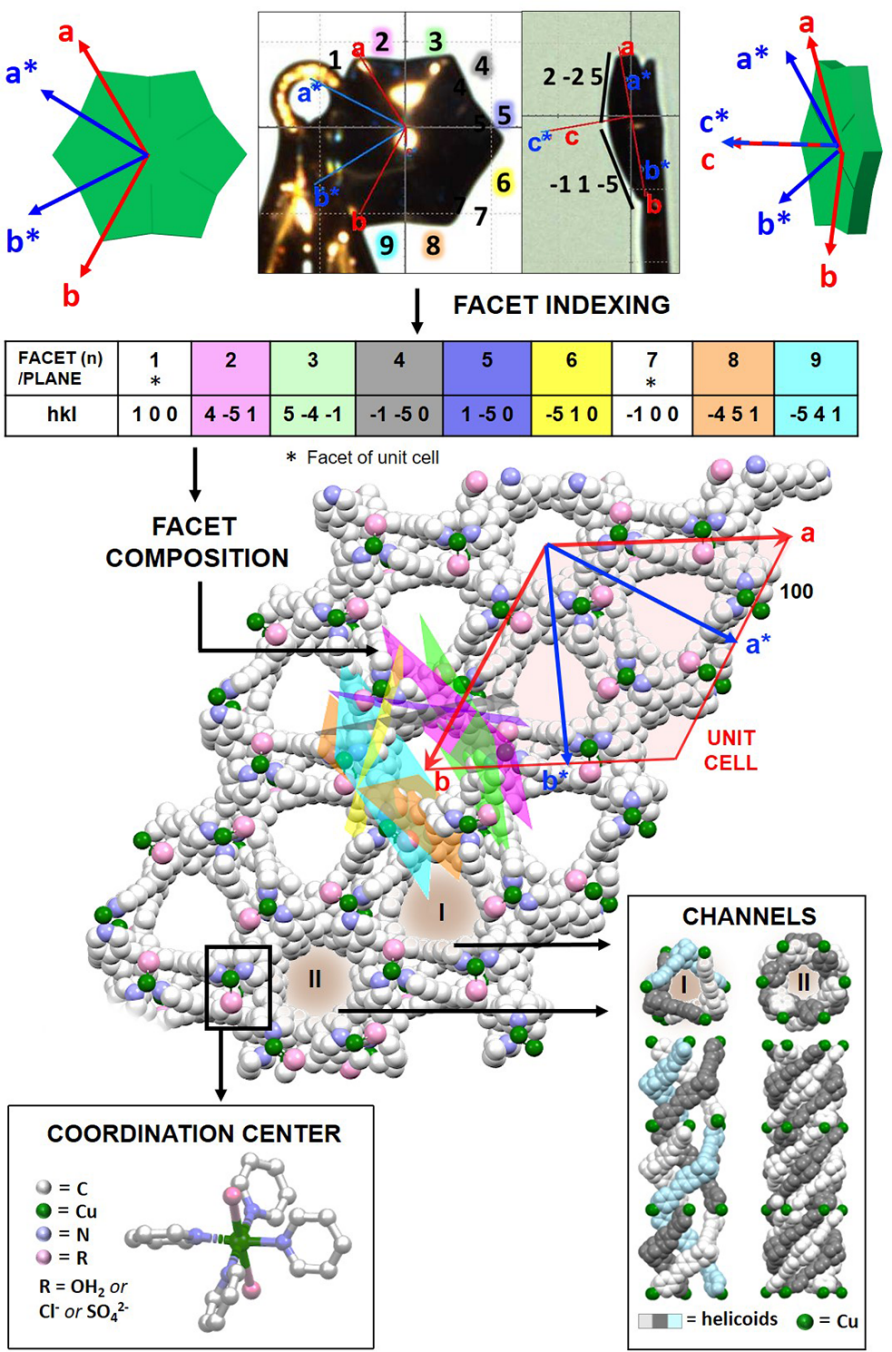
Linking the macroscopic and molecular levels. (top) Optical microscopy images of **CSTAR-SO_4_** mounted on a loop. (bottom) Crystallographic structure, viewed down the *c* axis, of the same **CSTAR-SO_4_** (CCDC 2117028). The optical microscopy images and crystal structures show unit cell axes in the real (red) and reciprocal (blue) spaces. The lateral facets are labeled by numbers and colors in the optical image on the left, and the experimentally determined *hkl* Miller index values are reported in the table. The corresponding planes are drawn on the crystallographic structure (see also Figure S7).

We hypothesize that a correlation exists between the complex morphology (concave polyhedron) and the open molecular packing. The orientation of the unit cell axes relative to the crystal morphology has been determined (Figures 4, S10–12). The *c*-axis is perpendicular to the stellate profile, whereas the *a* and *b* axes pass through two tips of the star. The concave faces deviate by ∼10° from a regular facet of a hexagonal prism. The 12 facets of three crystals were indexed using CrysAlisPro.^52^ The facets defining concave angles are related to each other by mirror symmetry, in agreement with the 6/*mmm* point group.

Using the indices summarized in the tables shown in Figures 4 and S10–12, we projected the corresponding Miller planes onto the crystallographic structures using Mercury. These planes are not the basal faces of a hexagonal unit cell. In contrast, the concave facets are at an angle of ∼10° to the unit cell edges and more closely correspond to the walls of the channels. The crystals have a low, inhomogeneous distribution of molecular density in the unit cell, with voids comprising ∼37% of the unit cell volume (calculated using the contact surface employing a spherical probe with a radius of 1.2 Å). As stated above, the 6-fold axis of the crystal runs through the center of a channel. Likewise, the larger 3-fold channels also center around special positions within the unit cell. Therefore, the low Miller-index crystal planes connecting these lattice points necessarily cut through the channels relatively devoid of the molecular material (unlike close-packed molecular crystals).

The stellate morphology of our crystals resembles that of cyclic twinned crystals observed in minerals^32−35^ and biominerals^34^ (Figure S13). In these cyclic twins (a form of polysynthetic twinning), morphological sectors are distinct crystallographic domains.^53^ The concave angles of these examples are inconsistent with single crystallinity.^31^ We have shown that in our crystals, both the multidomain appearance and the concave angles coexist with single crystallinity. The symmetry of the crystal morphology correlates with the expected point group, 622. However, the morphology is not among the expected forms (i.e., hexagonal trapezohedron, dihexagonal prism, hexagonal dipyramid, or hexagonal prism)^27^ for the point group 622, nor for any of the other point groups. The morphology can come about by variation in concentration during the crystallization,^54−56^ as shown for an isostructural Ni-based MOF (with a different morphology).^54^ Moreover, Oki et al. showed that varying the concentration resulted in concave organic structures.^56^

We observed that macroscopic features of these crystals are related to their molecular packing. This molecular–macro correlation is manifested both in the straight facets defining the form and the fine surface features. Our ligand systems have yielded not only the stellate crystals but previously reported single crystals having similar crystallographic structures (*P*622) and morphologies of remarkable complexity.^20,21,44^ The concave polyhedron shape single crystal is a new type of structure. The physicochemical properties of crystals are known to be related to their morphology.^57^ Crystals with high-index facets such as reported in this study might find applications in catalysis.^58^

## Supporting Information Available

Experimental details, materials, and methods. Crystal data and structure refinement, optical and scanning electron microscopy images, powder X-ray diffraction, and thermogravimetric analysis (PDF)
