# Supplementary material for: Crystallographic–Morphological Connections in Star Shaped Metal–Organic Frameworks
Source: J Am Chem Soc. 2022 Dec 12;144(50):22838–43. doi: 10.1021/jacs.2c09785 (PMC9782779; doi:10.1021/jacs.2c09785)
Supplement: Supplementary file 1 — ja2c09785_si_001.pdf [file ja2c09785_si_001.pdf]

Supporting Information

Crystallographic–Morphological Connections in Star Shaped Metal–Organic Frameworks

Maria Chiara di Gregorio,[1] Vivek Singh,[1] Linda J. W. Shimon,[2,*] Michal Lahav,[1,*] Milko E. van der Boom[1,*]

[1]Department of Molecular Chemistry and Materials Science,[2] Department of Chemical Research Support, Weizmann Institute of Science, Rehovot, 7610001 Israel. Email: linda.shimon@weizmann.ac.il; michal.lahav@weizmann.ac.il; milko.vanderboom@weizmann.ac.il

Materials

Chloroform ($CHCl_3$, ≥ 99.8%) and dimethylformamide (DMF, ≥ 99.8%) were purchased from Sigma Aldrich and J. T. Baker, respectively. $Cu(NO_3)_2·3H_2O$ (>98.0%) and $CuSO_4$ anhydrous (>99%) were purchased from Fluka and Riedel-de Haën, respectively. Reagents were used without further purification. Glass pressure tubes (Ace Glass, Inc., pressure tubes #15 with a plunger valve, PTFE bushing and FETFE® O-Ring, volume 50 mL) were cleaned in a base bath for two days. Subsequently, the tubes were washed with deionized (DI) water and dried in an oven for one day at 130°C. The ligand (1,3,5,7-tetrakis(4-((*E*)-2-(pyridin-4-yl)vinyl)phenyl)adamantane; **AdDB**) was prepared according to a literature procedure.[S1]

Preparation of the crystals: CSTAR-NO_3 and **CSTAR-SO_4**

The metal salt ($Cu(NO_3)_2·3H_2O$: 5.0 mg, 21 mmol or $CuSO_4$: 5.0 mg, 31 mmol) was dissolved in DMF (1.6 mL for $CuSO_4$; 1.1 mL for $Cu(NO_3)_2·3H_2O$). A mixture of $CHCl_3$ (1.0 mL) and DMF (2.0 mL) in a glass vial (20 mL) was sonicated (33-40 KHz frequency) for 1.5 h in an ice bath. Subsequently, this solvent mixture was used immediately to dissolve **AdDB** (3.0 mg, 3.5 mmol). The solution of **AdDB** and 1.0 mL of the solution of the metal salt were mixed in a glass pressure tube (the final concentrations of **AdDB** and the metal salt were 0.9 mM and 1.8 mM, respectively). Then, the tube was sealed and heated for 48 h in an oven at 105°C. Subsequently, the temperature of the controller was decreased every hour by 10°C. **CSTAR-NO_3** was isolated as a green powder by centrifugation, washed with ethanol and re-dispersed in 1.0 mL ethanol. **CSTAR-SO_4** appeared both as green crystals at the bottom of the tube and as a greenish powder. Powder X-ray diffraction (PXRD), thermogravimetric analysis (TGA), elemental analyses has been provided in the supporting information (**Figure S12-S13, Table S3**).

Scanning electron microscopy (SEM)

SEM measurements were performed using HRSEM ULTRA-55 ZEISS instruments at an EHT voltage ranging from 1-1.5 kV. Images were collected in secondary electron mode by using an Everhart-Thornley detector. Samples of **CSTAR-NO_3** were prepared by drop-casting a dispersion in ethanol on a silicon substrate. Two sample-preparation procedures were used on a carbon tape covering the SEM stub for the imaging of **CSTAR-SO_4**: (a) the reaction mixture was drop-casted and then the solvent was allowed to evaporate, (b) the reaction mixture was drop-casted on a microscope slide, then dried, and the residue was washed with ethanol, dispersed in ethanol and drop-casted on the carbon tape.

Light microscopy images

Light microscopy images were obtained with Nikon's Eclipse E600 and Nikon Eclipse LV100ND microscopes at 20X magnification. The latter microscope is equipped with DeltaPix 2001-2022 v6.5.3 software that allows for extended focus imaging.

Powder X-ray Diffraction (PXRD)

The PXRD measurements were performed in reflection geometry by using a TTRAX III (Rigaku, Japan) diffractometer. The diffractometer is equipped with a rotating Cu anode operating at 50 kV and 200 mA. A bent graphite monochromator and a scintillation detector were aligned in the diffracted beam. $\theta/2\theta$ scans were performed under specular conditions in the Bragg-Brentano mode with variable slits. The MOFs dispersion was drop casted on a silicon plate and allowed to dry at the air before the measurement. PXRD spectra were collected from 5 to 25 degrees with step size of 0.025 degrees. These experimental PXRD patterns were analyzed by using Jade 2010 software (Materials Data, Inc.). The lattice parameters were refined using the Whole Pattern Fitting/Rietveld refinement module of Jade 2010.

Single Crystal X-ray (SXRD)

SXRD data were collected both at the synchrotron source (ESRF ID-29) of the European Synchrotron Radiation Facility (ESRF) and using home-lab Rigaku XtaLab^{Pro} or Synergy diffractometers. Beamline ID-29 was equipped with a Pilatus 6M. The Rigaku XtaLab^{Pro} X-ray diffractometer was equipped with a 4-circle Kappa goniometer, a Pilatus Dectris 3R S200K-A detector, and a micro-focus sealed tube with microCMF-VHF. The Rigaku Synergy was equipped with a Pilatus Dectris 300K CdTe detector. The Rigaku Synergy-R was equipped with a HyPix ARC150° detector. The data were collected with λ = 0.700 Å (ID-29) and CuK$_{\alpha1}$ radiation, λ = 1.5418 Å (Rigaku XtaLab^{Pro} and Synergy-R) and MoK$_{\alpha1}$ λ = 0.71073 Å (Rigaku Synergy).

The crystals were placed in Hampton Paratone oil, mounted on a MiTeGen loop and plunged into liquid nitrogen to flash freeze. The data were collected at 100 K with Oxford Cryostream. The crystals analyzed at the synchrotron were transported frozen in a Taylor-Wharton CX100 dry shipper. Data collection and reduction for the synchrotron ID-29 data were done using MXCube, and the EDNA automated data processing pipeline with XDS. The structures were solved by direct methods using SHELXT implemented with the Olex2 software GUI.[S2]

The structures were refined by full-matrix least-squares methods on F^2 with SHELXL. All non-hydrogen atoms were refined with anisotropic displacement coefficients. Hydrogen atoms were placed in calculated positions, assigned isotropic displacement coefficients, U(H) = 1.2U(C) or 1.5U (C-methyl), and their coordinates were allowed to ride on their respective carbons.

Data collection, reduction and analysis for the XtaLab^{Pro} laboratory data were performed with the CrysAlisPro software package (version 1.171.39.22a, Rigaku OD, 2018). Data collection, reduction and analysis for the Synergy laboratory data were performed with the CrysAlisPro software package (version 1.171.40.80a, Rigaku OD, 2018). The crystal structures were solved by direct methods using SHELXT 2016/4. All non-hydrogen atoms were further refined by SHELXL with anisotropic

displacement coefficients. Hydrogen atoms were placed in calculated positions and refined in riding mode on the respective carbon atoms. The Platon SQUEEZE protocol was applied for all the structures. Mercury CSD 3.10.2 and PLATON software were used for graphics.[S3] The crystallographic data and refinement parameters are summarized in **Table S1**. All crystals have the same rare space group (*P*622). There are 43 structures with this space group in the database of the CCDC of which 26 have been reported by us.[S4-S7]

The Ewald sphere's projections were produced by CrysAlis^{Pro} from data sets collected on the Rigaku XtaLab^{Pro} or Synergy systems. These home-laboratory system generators have microfocus X-ray tubes with beams approximately 100 μm in cross-section; such a beam is large enough to expose the entire crystal simultaneously so that the diffraction data shown plotted in the Ewald spheres is from all the sections of the stars. The Cumulative Intensity statistics were plotted within Olex2.[S8] The Laue symmetry statistics and twinning analysis were produced in the XPREP module of SHELX. Details of the crystal structure analysis are reported in the table below.

Indexing of the facets and twinning analysis

The stellate crystals on the Rigaku Synergy or Synergy R were measured and the data were fully processed with CrysAlis^{PRO} *1.171.40*, software package (Rigaku OD, 2018). Prior to the X-ray measurement, a 360º video of the mounted crystal was taken for absorption corrections and crystal morphology determination. The approximate magnification of this video system is x450, allowing us to clearly view the crystal (**Figures 4**, **S6** and **S7**). The video camera of XtaLab^{Pro} is unfortunately of insufficient magnification for this purpose. After the unit cell and space group were determined and the structure solution completed (with Shelxl and Shelxt as implemented in Olex2 Gui), the pre-recorded video frames were used for crystal face indexing routine. The unit cell axes vis-à-vis the crystal were determined (**Figures 4**, **S6** and **S7**). The crystal shape routine of CrysAlis^{Pro} allows for the

placement of the best planes the crystal faces, and the approximate Miller indices (*hkl*) of the faces were indexed where the estimated low integer hkl option was used. It should be noted that the program routine does not allow for the drawing of the unexpected re-entrant angles. Some faces were obscured by the loop or by the meniscus of the cryoprotectant oil. The best planes were identified for each of the faces, where possible. The star morphology appears to comprise vicinal faces that cause deviation from the traditional 1st-order hexagonal prism. In our case, as in other 1st-order prisms, the unit cell axes emerge from the vertices between the two prism faces and the Miller indexes;[S9] if it were not for the concavity, the faces

The existence of the unusual star-shaped crystal combined with a rare, high-symmetry space group raised suspicions of possible crystal twinning. By definition, 'a twin consists of two or more single crystals of the same species but in different orientations.[S10] The twin components are grown together in a shared surface called the *composition surface* or *twin interface*.

The twinning of crystals, on the macroscopic scale, sometimes manifests as the presence of re-entrant crystal angles.[S11] When the twinning interfaces are not parallel to each other then they develop as cyclic twins, which can resemble stars. However, the presence of morphological irregularities is in itself not a sufficient indicator. While concave crystal faces do not always indicate twinning, it is certainly a warning sign.[S10] On the atomic level, twinning, as with other crystal imperfections, might manifest as certain pathologies such as split reflections and deviation from the expected intensity statistics of diffraction data.[S12] However, some forms of twinning can be particularly difficult to determine and not evident until full data sets are analyzed. In particular, *merohedral twinning* is a type of twinning where the lattices of two or more distinct domains coincide exactly.[S13] As the real space lattices coincide, the reciprocal lattices of the domains will exactly overlap and the resulting diffraction pattern will appear normal. This type of twinning can occur in space groups of high symmetry, e.g., tetragonal, trigonal, hexagonal or cubic, where more than one Laue symmetry is possible. In such

cases, the additional rotational symmetry operator of the twinning exactly superimposes parts of the diffraction pattern, causing the appearance of higher symmetry, i.e., the twinning adds symmetry. The true Laue symmetry (point symmetry) of the crystal is lower than that of the lattice.[S14] The hexagonal space group *P*622 can be a result of the merohedral twinning of the lower symmetry trigonal space group *P*321. Therefore, it is imperative for our analysis that this possibility be checked and definitively confirmed or denied. As explained above, since merohedral twinning will not be evident from the diffraction pattern, the intensity distribution of the complete data set needs to be examined.[S15]

One such widely used analysis involves the identification of twins from intensity statistics. The values of the $<|E_2-1|>$ can be plotted and are known to differ for centrosymmetric vs. non-centrosymmetric structures. The theoretical statistical value for centric structures is 0.938,[S11] and that of acentric structures 0.736. Twinning results in a diffraction pattern of higher symmetry and yields a hypo-non-centrosymmetric intensity distribution.[S16] The cumulative distribution function N(z) gives the fraction of reflections whose relative intensities are less than z, where z is the normalized reflection intensity obtained by dividing each individual measurement by the average value for its resolution shell.[S10] When the plot of the cumulative intensity distribution N(Z) for acentric and centric data is plotted, the shape of the plot is a diagnostic. The plot for an un-twinned crystal appears exponential due to the existence of a small number of very weak or very strong reflections, while for a twinned crystal, the plot appears sigmoidal. The sigmoidal shape arises because a portion of the non-equivalent reflections in a twinned crystal will overlap exactly;[S17] some of the very strong reflection intensities become averaged with very weak reflections and thus the distribution of reflection intensities N(z) will vary from the theoretical.[S18] The cumulative intensity test for star-shaped crystals CIF v440 (CCDC 2117030) and CIF v339 (CCDC 2009649) was calculated in the Olex2 software package and are presented in **Figure 3B**. The plots clearly show the exponential shape indicative of un-twinned crystals.

Laue analysis

Perfect twinning and the additional twin laws can result in a diffraction pattern and space group with a higher apparent symmetry than the true crystal symmetry. Twinning can also be determined by comparing the R_{int} values of the true Laue group with those of the apparent Laue group. The R_{int} (as calculated in Shelx XPREP) will be lower for the true space group. The results of this analysis are shown below **Figure 3C**. As can be seen, in all the cases, the R_{int} for the high symmetry *622* is lower than that of the lower symmetry Laue group. This indicates that the higher symmetry of group *P*622 is indeed true and not an artifact of twinning in lower symmetry. Combined with the results of the cumulative intensity plots, we are confident that our unusual stellate crystals are single.

Table S1. Crystal data and structure refinement parameters of **CSTAR-NO$_3$**.

CIF file name	V241	V339
CCDC	2009648	2009649
Crystal description	Star	Star
Source	ESRF ID29	Rigaku XtaLabPro
Empirical formula	$C_{62}H_{52}CuN_4O_2$	$C_{62}H_{52}CuN_4O_2$ + 2(O)
Formula weight (g/mol)	948.61	980.61
Temperature (K)	100	100
Wavelength (Å)	0.7	1.54184
Crystal system	hexagonal	hexagonal
Space group	*P622*	*P622*
a (Å)	25.510(4)	25.3956(12)
b (Å)	25.510(4)	25.3956(12)
c (Å)	18.120(4)	18.1190(7)
α, β, γ°	90, 90, 120	90, 90, 120
Volume (Å^3)	10212(4)	10120.0(10)
Z	6	6
Density calculated (Mg/m^3)	0.925	0.965
Absorption coefficient (mm^{-1})	0.343	0.770
F(000)	2982	3078
Theta range for data collection (°)	0.908 to 24.940	4.020 to 48.00
Reflection collected (Unique)	60570 (6188)	10129 (3155)
R_{int}	0.039	0.0432
Completeness %	99.0	99.5
Data/restraints/parameters	6188/0/313	3155 /11/ 322
Goodness-of-fit on F^2	1.062	1.091
Final R [I>2σ(I)]	R1=0.0712 wR2=0.2166	R1=0.0808 wR2=0.2274
R (all data)	R1=0.0747 wR2=0.2228	R1=0. 0916 wR2=0.2392
Largest diff. peak and hole (e$\cdot$ Å^{-3})	0.855 and -0.367	0.474 and -0.322
Flack parameter	0.043(6)	0.09(4)
Channels' helicity	M	P

Table S2. Crystal data and structure refinement parameters of **CSTAR-SO_4**. No π-π interactions have been found. CCDC 2117028 has been checked with the Olex2 software geometry module.

CIF file name	V412	V440	V465	V466	V562
CCDC	2117031	2117030	2117028	2117029	2214170
Crystal description	Star	Star	Star	Star	Single point
Source	Rigaku Synergy	Rigaku Synergy	Rigaku Synergy-R	Rigaku Synergy-R	Rigaku Synergy-R
Empirical formula	$C_{62}H_{52}ClCuN_4O_4S$ + 2(O)	$C_{62}H_{52}CuN_4O_2$	$C_{62}H_{52}Cl_2CuN_4$	$C_{62}H_{52}Cl_2CuN_4$	$C_{62}H_{52}Cl_2CuN_4$
Formula weight (g/mol)	1080.12	948.63	987.51	987.51	987.51
Temperature (K)	100	100	100	100	100
Wavelength (Å)	0.71073	0.71073	1.54184	1.54184	1.54184
Crystal system	hexagonal	hexagonal	hexagonal	hexagonal	hexagonal
Space group	*P622*	*P622*	*P622*	*P622*	*P622*
a (Å)	25.4975(9)	25.4680(6)	25.4447(4)	25.3788(10)	25.5173(9)
b (Å)	25.4975(9)	25.4680(6)	25.4447(4)	25.3788(10)	25.5173(9)
c (Å)	18.1958(5)	18.2135(5)	18.1941(2)	18.2533(5)	18.1389(5)
α, β, γ°	90, 90, 120	90, 90, 120	90, 90, 120	90, 90, 120	90, 90, 120
Volume ($Å^3$)	10244.6(8)	10230.9(4)	10201.3(3)	10181.5(8)	10228.5(8)
Z	6	6	6	6	6
Density calculated (Mg/m^3)	1.051	0.924	0.964	0.966	0.944
Absorption coefficient (mm^{-1})	0.434	0.356	1.426	1.429	1.422
F(000)	3078	2982	3090	3090	3090
Theta range for data collection (°)	1.597 to 32.030	1.76 to 28.69	3.474 to 75.106	3.483 to 64.852	3.464 to 67.684
Reflection collected (Unique)	106742 (11857)	177201 (8832)	45828 (6988)	23203 (5536)	26692 (6250)
R_{int}	0.0412	0.0315	0.0205	0.0241	0.0301
Completeness %	99.2	99.7	99.2	97.1	99.6
Data/restraints/parameters	11857 /51/ 387	8832/56/332	6988/0/314	5536/22/314	6250/20/296
Goodness-of-fit; F^2	0.998	1.012	1.021	1.081	1.032
Final R [I>2σ(I)]	R1=0.0749 wR2=0.2066	R1=0.0620 wR2=0.1840	R1=0.0673 wR2=0.2019	R1=0.0787 wR2=0.2320	R1=0.0820 wR2=0.2305
R (all data)	R1=0. 0950 wR2=0.2195	R1=0.0719 wR2=0.1952	R1=0.0698 wR2=0.2061	R1=0.0864 wR2=0.2439	R1=0.0977 wR2=0.2500
Largest diff. peak and hole ($e^{\cdot}$ $Å^{-3}$)	0.697 and -0.569	0.943 and -0.436	0.951 and -0.359	1.174 and -0.521	1.042 and -0.390
Flack parameter	0.010(5)	0.04(3)	0.10(4)	0.03(5)	0.13(5)
Channels' helicity	M	M	P	M	M

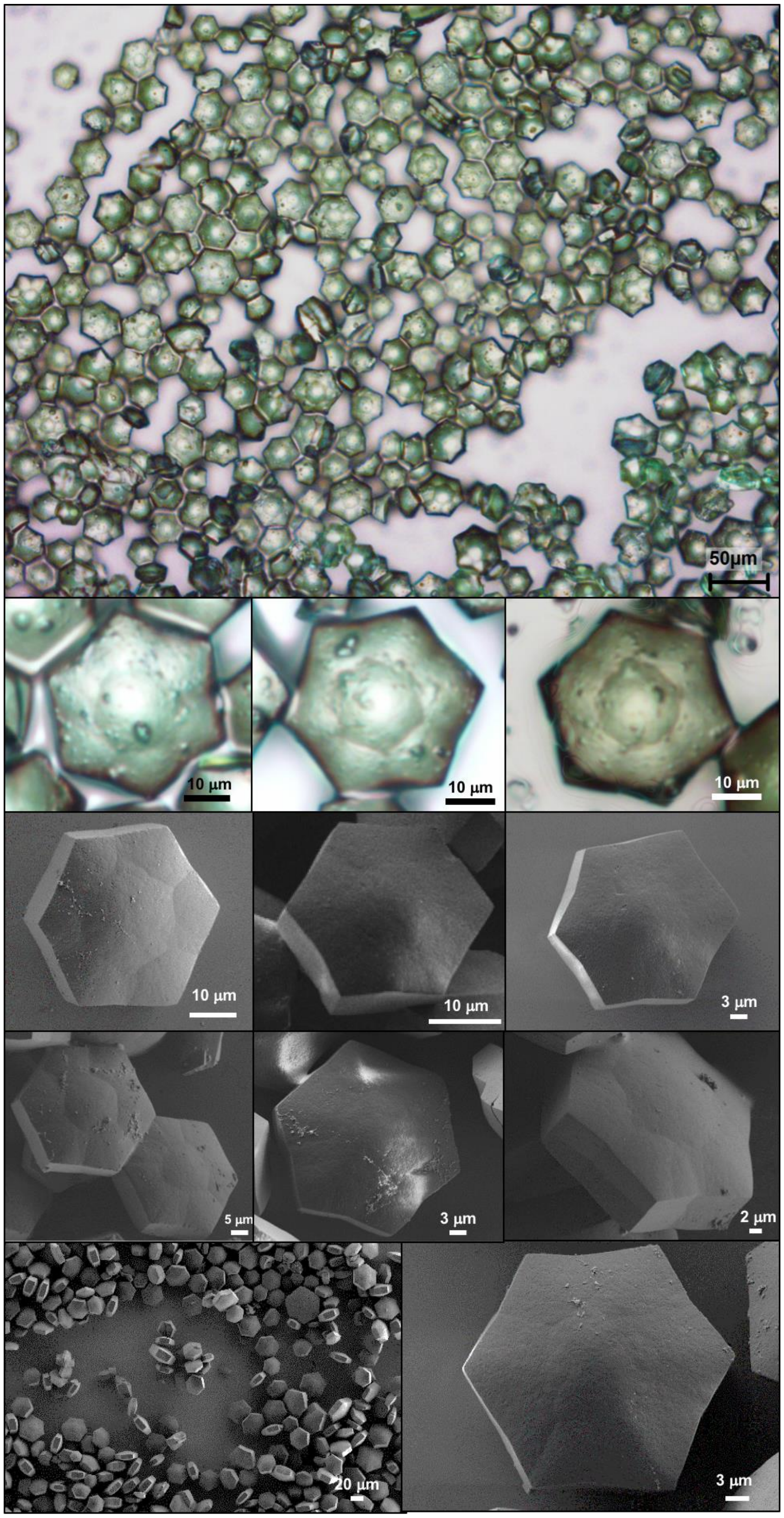

Figure S1. Light microscopy (top) and scanning electron microscopy (bottom) images of **CSTAR-NO_3**.

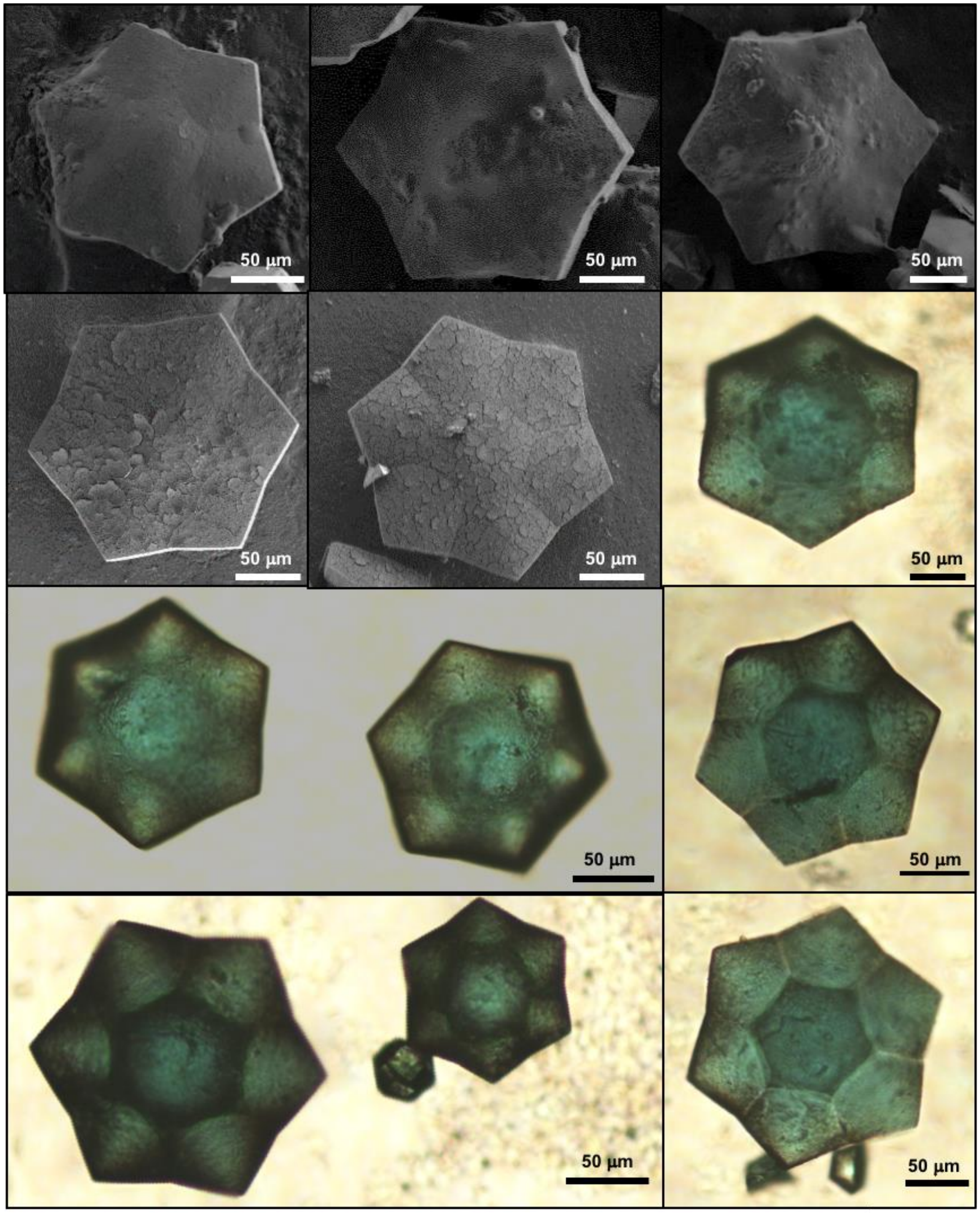

Figure S2. Scanning electron microscopy and light microscopy images of **CSTAR-SO_4**.

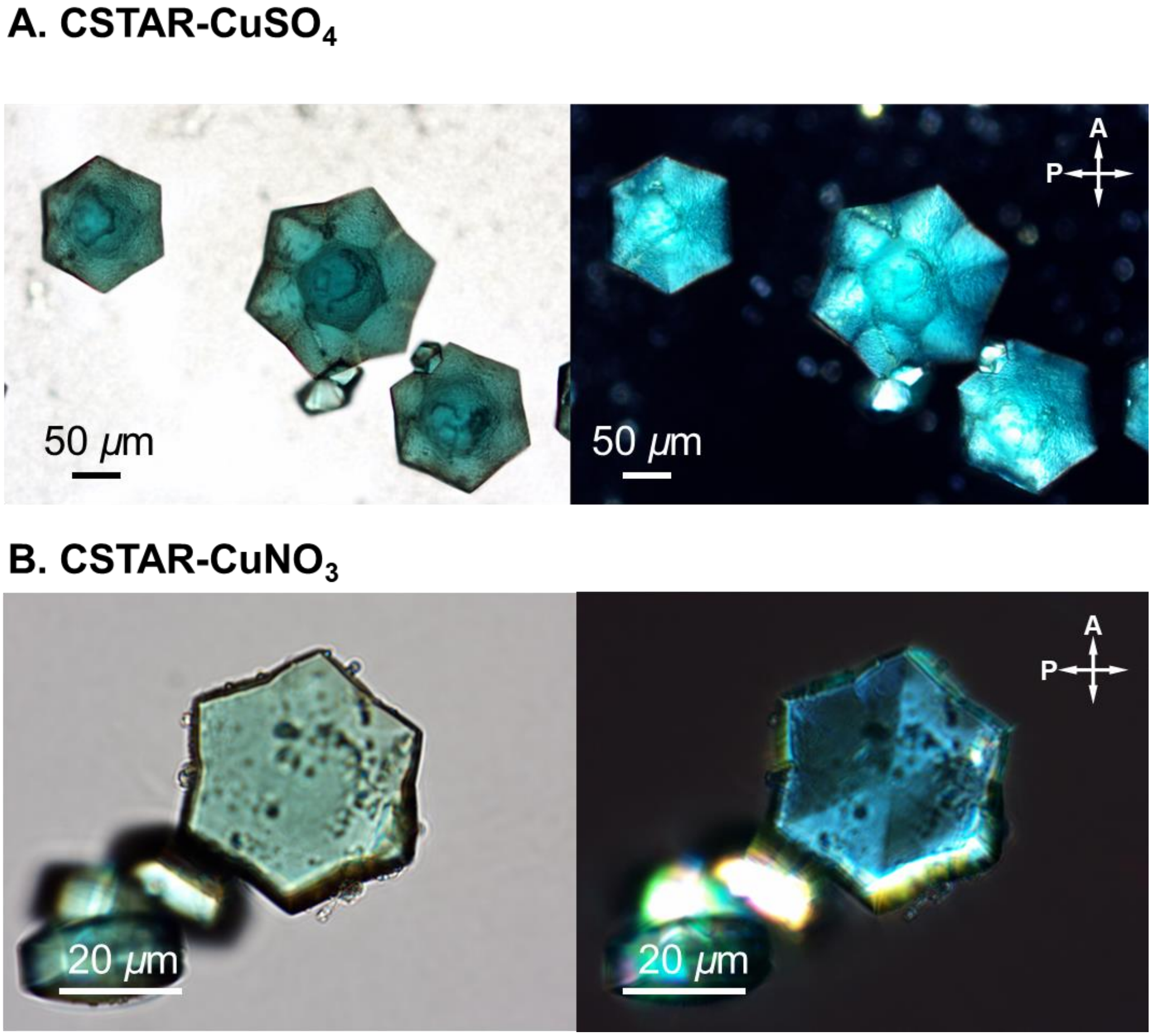

Figure S3. Optical microscopy images of crystals without polarizer (left) and with polarizer (right). A = Analyzer, P = Polarizer.

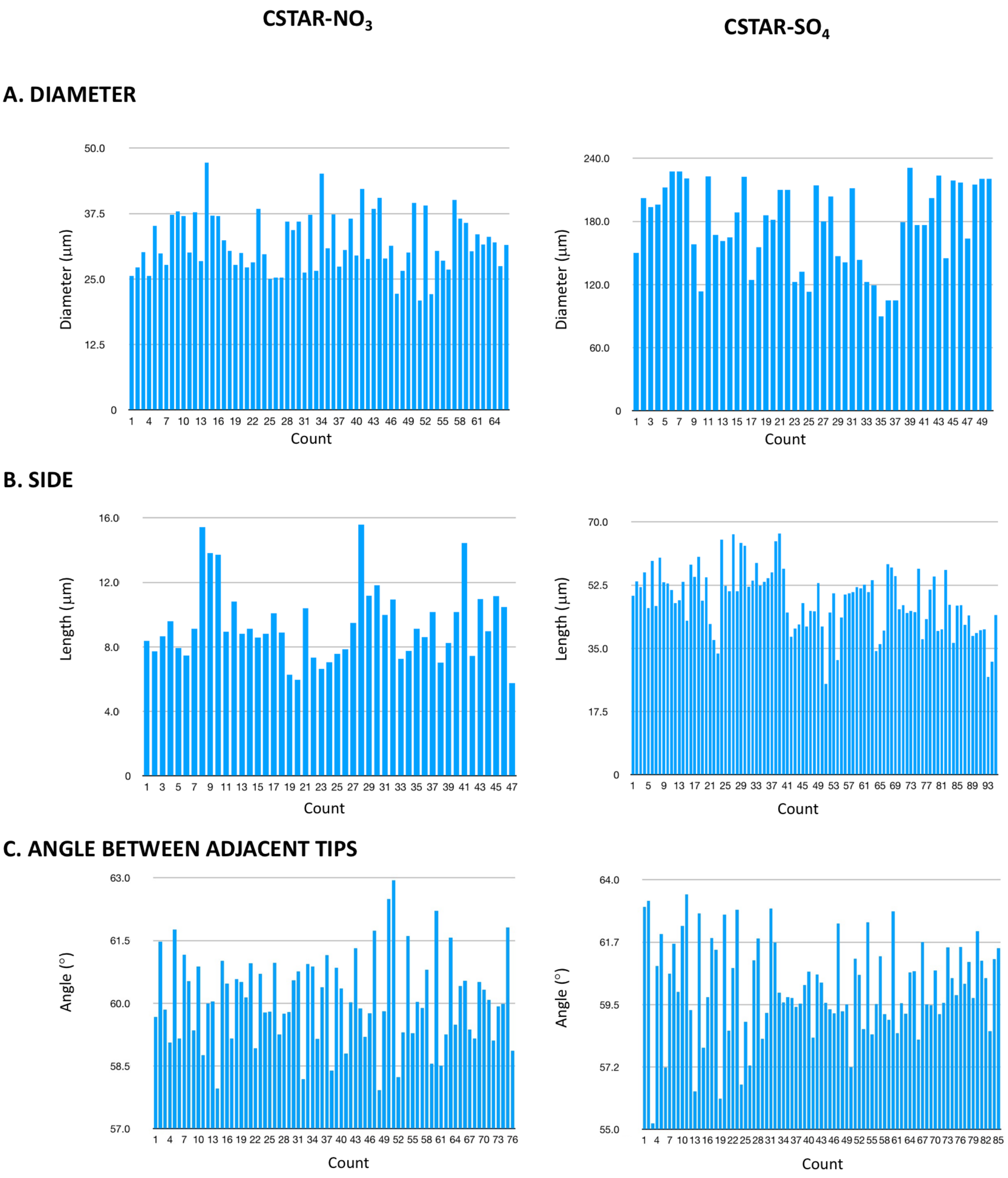

Figure S4. Dimensions of **CSTAR-NO$_3$** (left) **CSTAR-SO$_4$** (right) obtained from microscopy images.

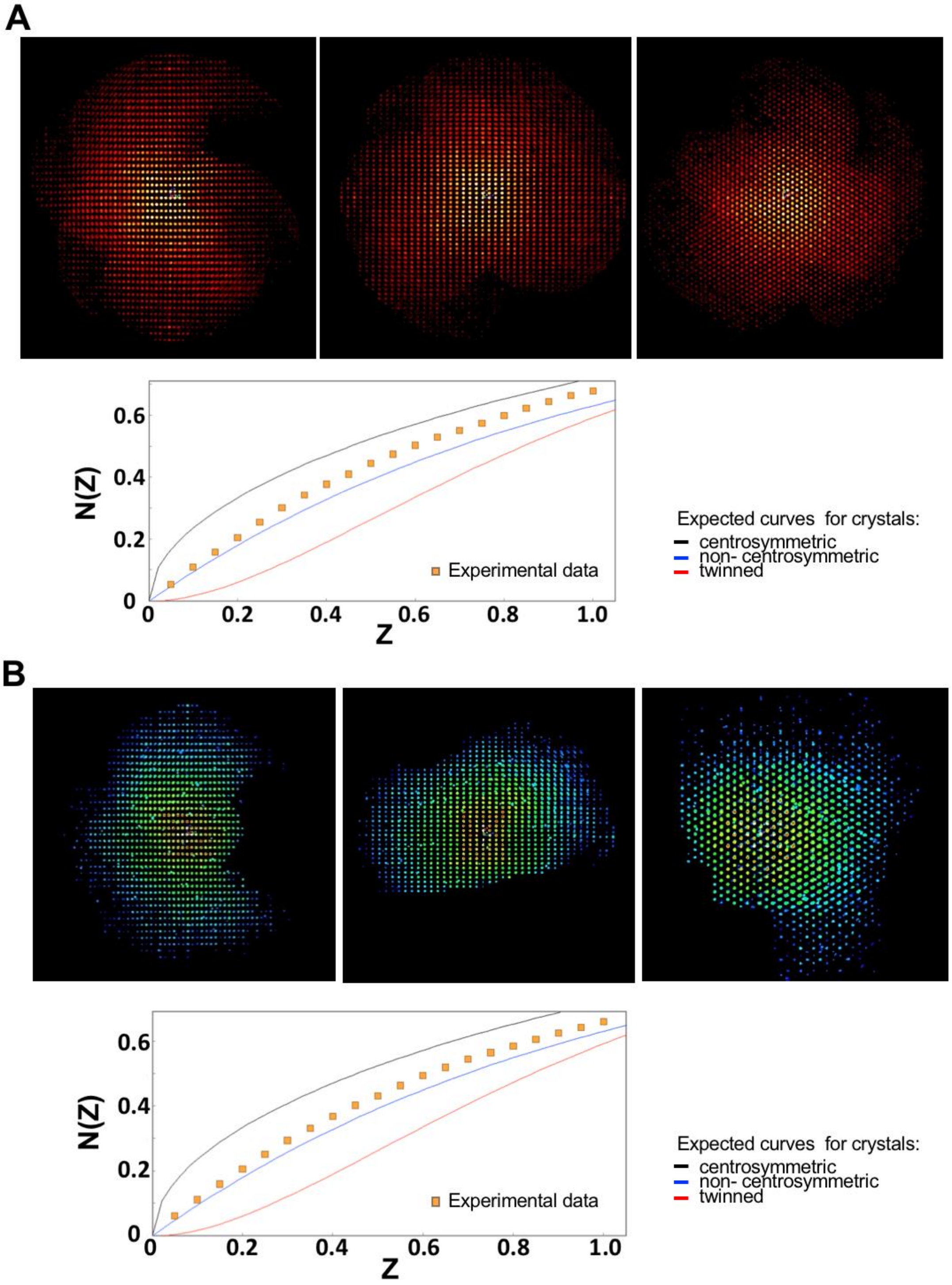

Figure S5. Ewald sphere projections and cumulative intensity distribution plots of two **CSTAR-SO_4** (CIF v465 CCDC 2117028 and CIF v466 CCDC 2117029 for A and B, respectively) crystals.

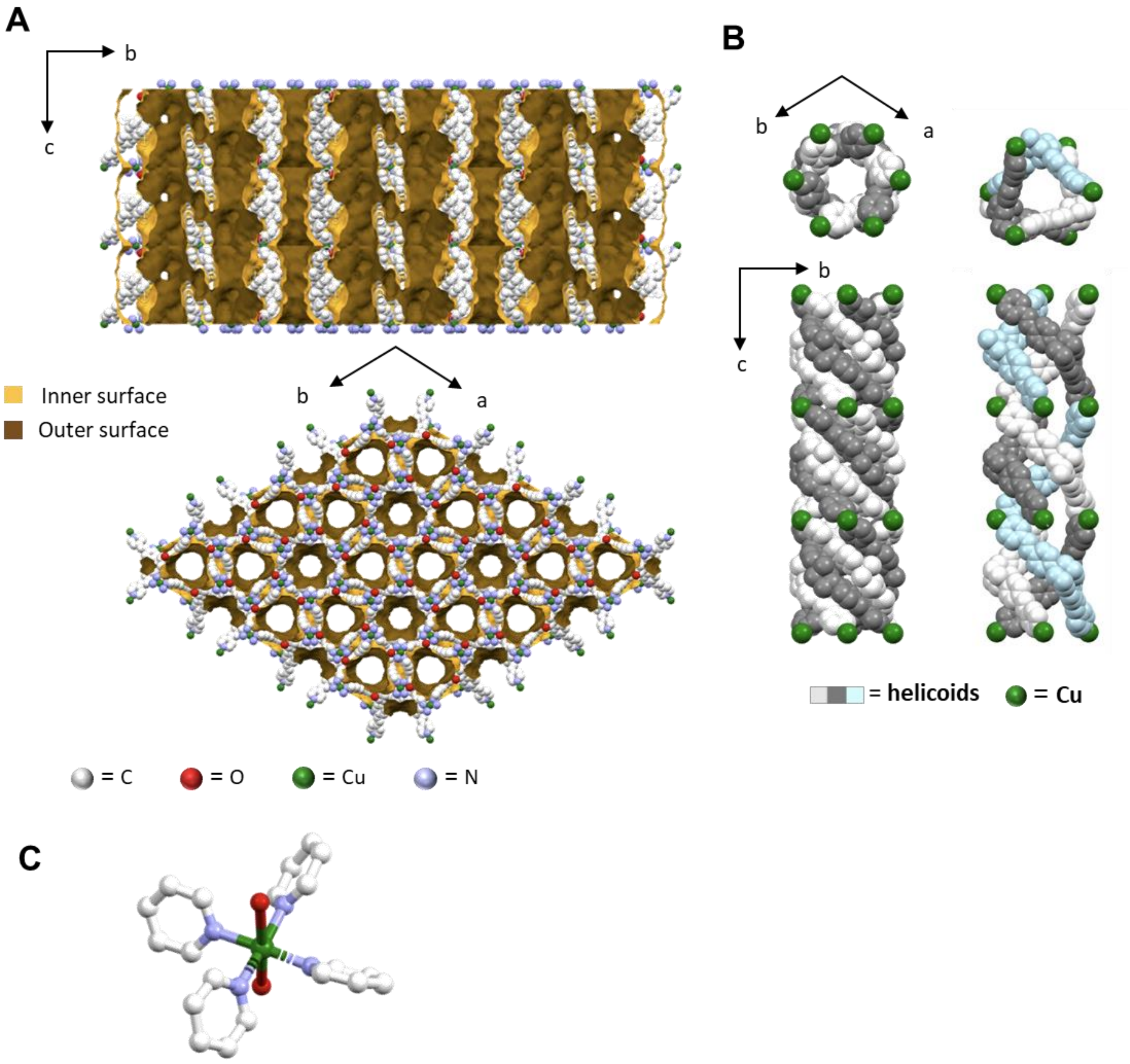

Figure S6. Single-crystal X-ray data of **CSTAR-NO_3** (CIF 241 CCDC 2009648, **Table S1**). (A) Connolly surface representation of the crystal structure down the *a* and *c* axes. (B) Top and lateral views of the channels with hexagonal and triangular geometries. (C) Structure of the coordination center.

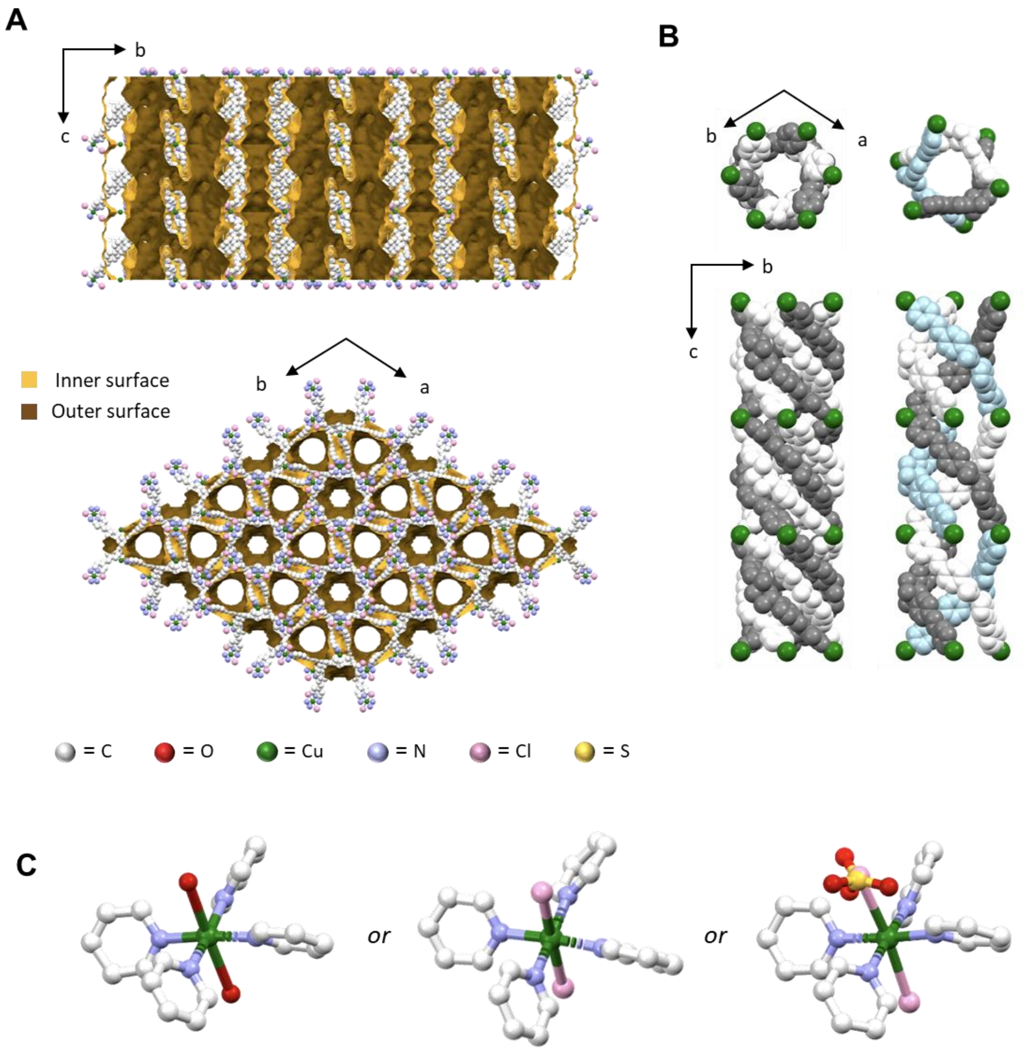

Figure S7. Single-crystal X-ray data of **CSTAR-SO_4** (**Table S2**). (A) Connolly surface representation of the crystal structure down the *a* and *c* axes. (B) Top and lateral views of the channels with hexagonal and triangular geometries. (C) Structure of the coordination center. In the axial position, different groups were detected, depending on the analyzed crystals: Cl in both axial positions were detected for v466 and v465 (CCDC 2117029 and CCDC 2117028, respectively); Cl and SO_4 moieties were detected for the crystal v412 (CCDC 2117031); O belonging to water molecules were detected for v440 (CCDC 2117030).

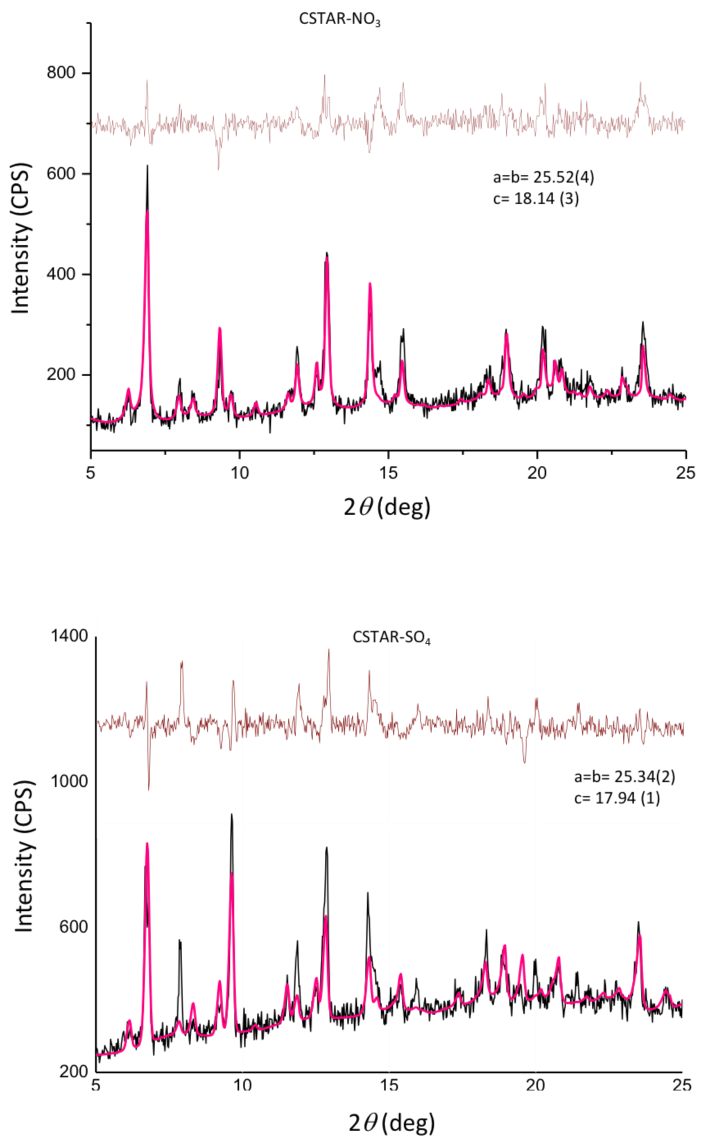

Figure S8. Experimental powder X-ray diffraction (PXRD) spectra of **CSTAR-NO$_3$** (top) and **CSTAR-SO$_4$** (bottom). The experimental PXRD spectrum was fitted (purple line) by using the single-crystal X-ray data (CIF v241 CCDC 2009648 and CIF v412 CCDC 2117031for **CSTAR-NO$_3$** and **CSTAR-SO$_4$** respectively). The difference between the fitted and experimental curves is shown at the top of the spectra (brown lines). The goodness-of-fit is 1.48 and 1.91 for the analysis of the spectra of **CSTAR-NO$_3$** and **CSTAR-SO$_4$**, respectively. The best fitting values of the unit cell dimensions are shown. Experimental intensity variations due to a preferred orientation was considered in the fit by using spherical harmonic functions (Jade 2010).

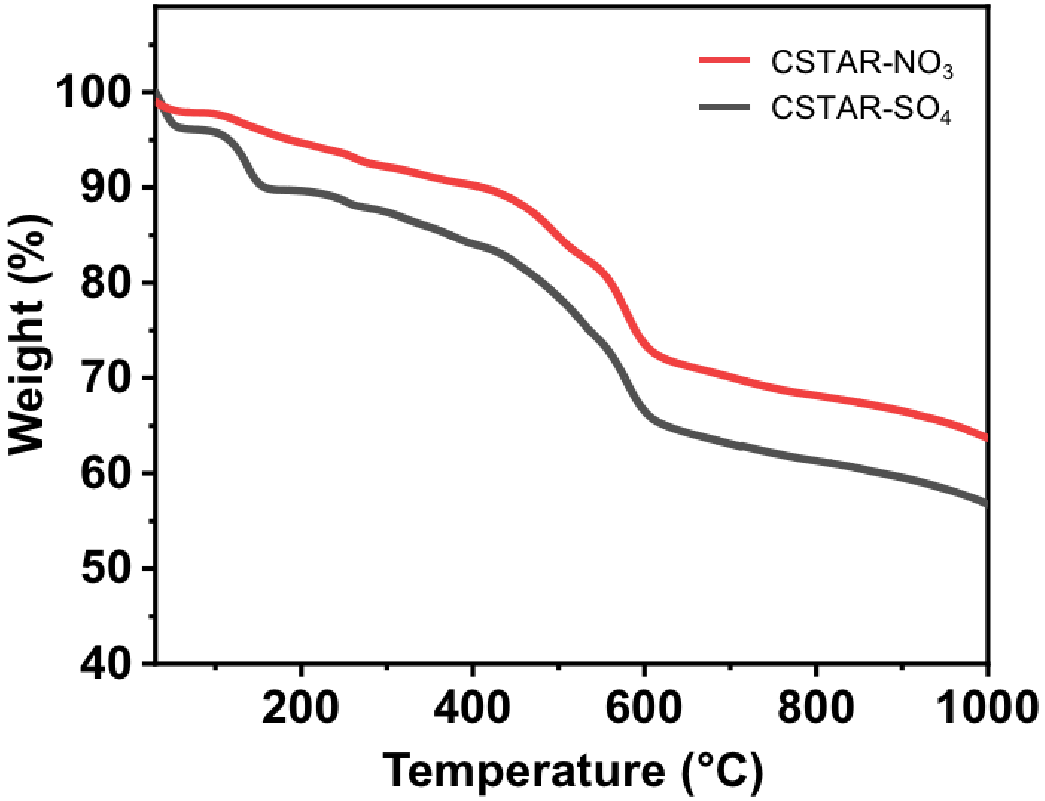

Figure S9. Thermogravimetric (TGA) curves under N_2 of **CSTAR-NO_3** (red) and **CSTAR-SO_4** (black) showing a similar thermal stability and loss of solvents.

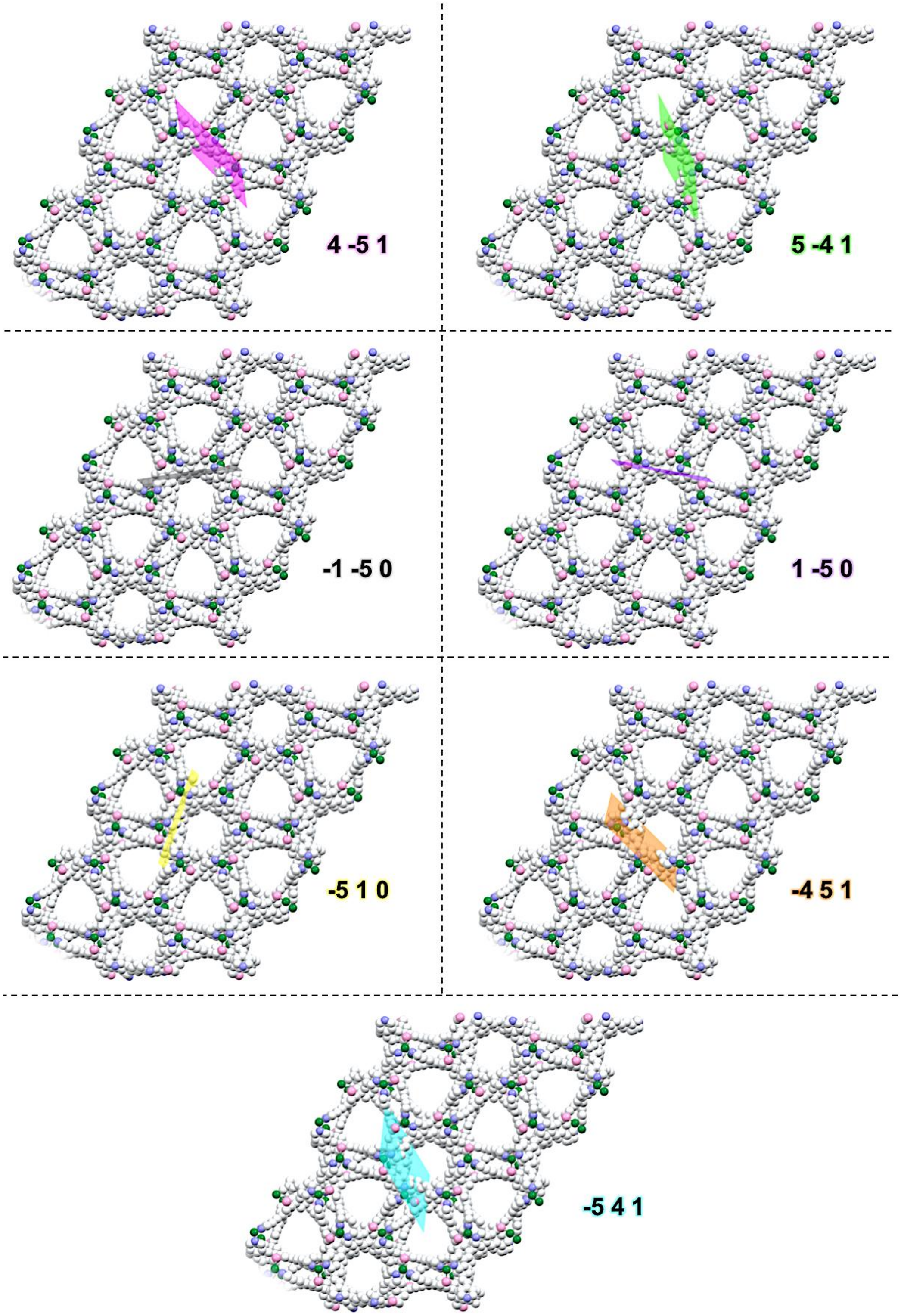

Figure S10. The planes obtained by indexing the lateral facets of **CSTAR-SO_4** (CCDC 2117028, **Fig. 4**) are shown separately on the crystal structure, view down the *c* axis.

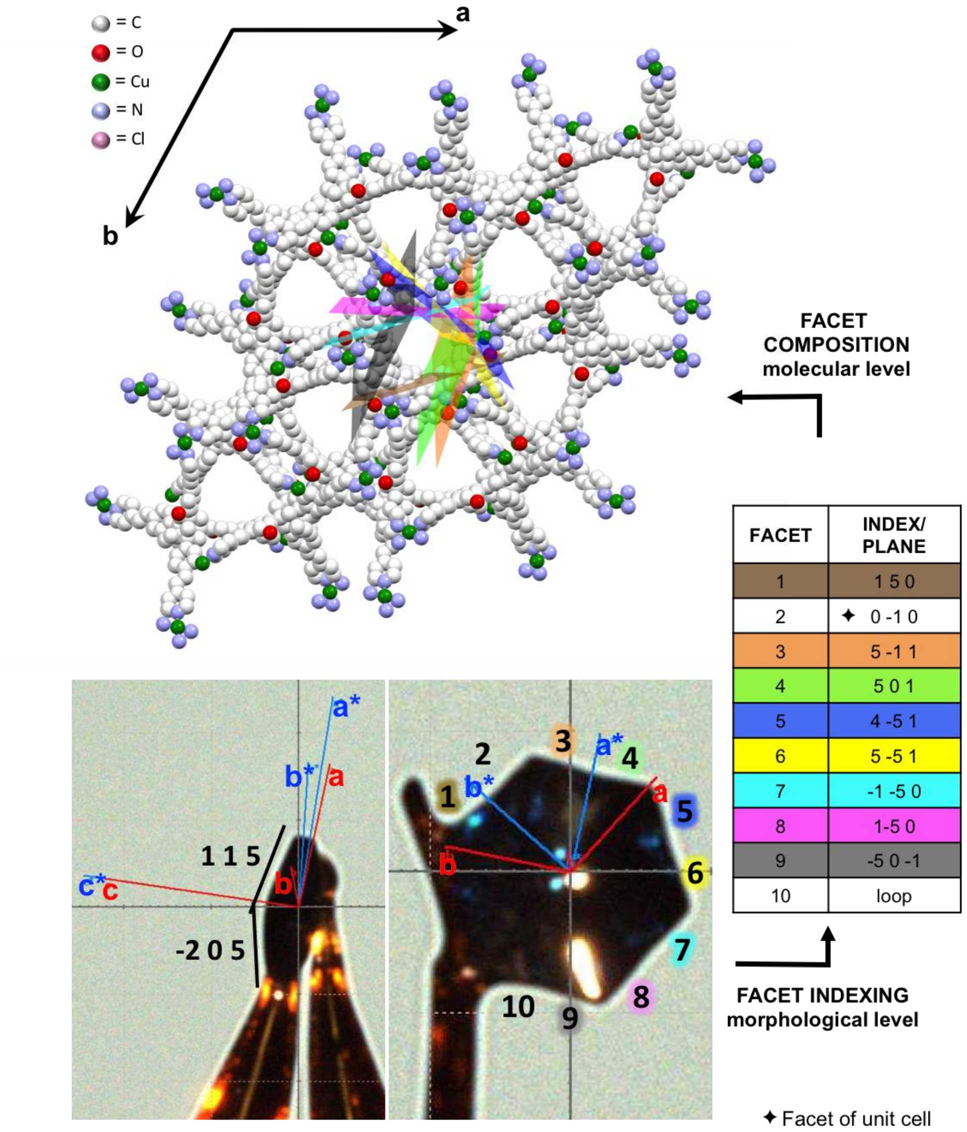

FACET	INDEX/ PLANE
1	1 5 0
2	✦ 0 -1 0
3	5 -1 1
4	5 0 1
5	4 -5 1
6	5 -5 1
7	-1 -5 0
8	1-5 0
9	-5 0 -1
10	loop

Figure S11. Crystallographic structure down the *c* axis of **CSTAR-SO$_4$** (CIF v440, CCDC 2117030) (top). Optical microscopy images related to the side and top view of the same **CSTAR-SO$_4$** crystal mounted on a MiTeGen loop (bottom). Based on the crystal structure, the facet indexes and the orientation of the axes vs. the morphology of the mounted crystal were determined by CrysAlisPro 1.171.40.57a (Rigaku OD, 2019). The axes in the real (red) and reciprocal (blue) spaces are reported in the optical images. The top facets are marked in black and indexed in the optical image on the left. The lateral facets are labelled by numbers in the optical image on the right, and the relative *hkl* values are reported in the table.

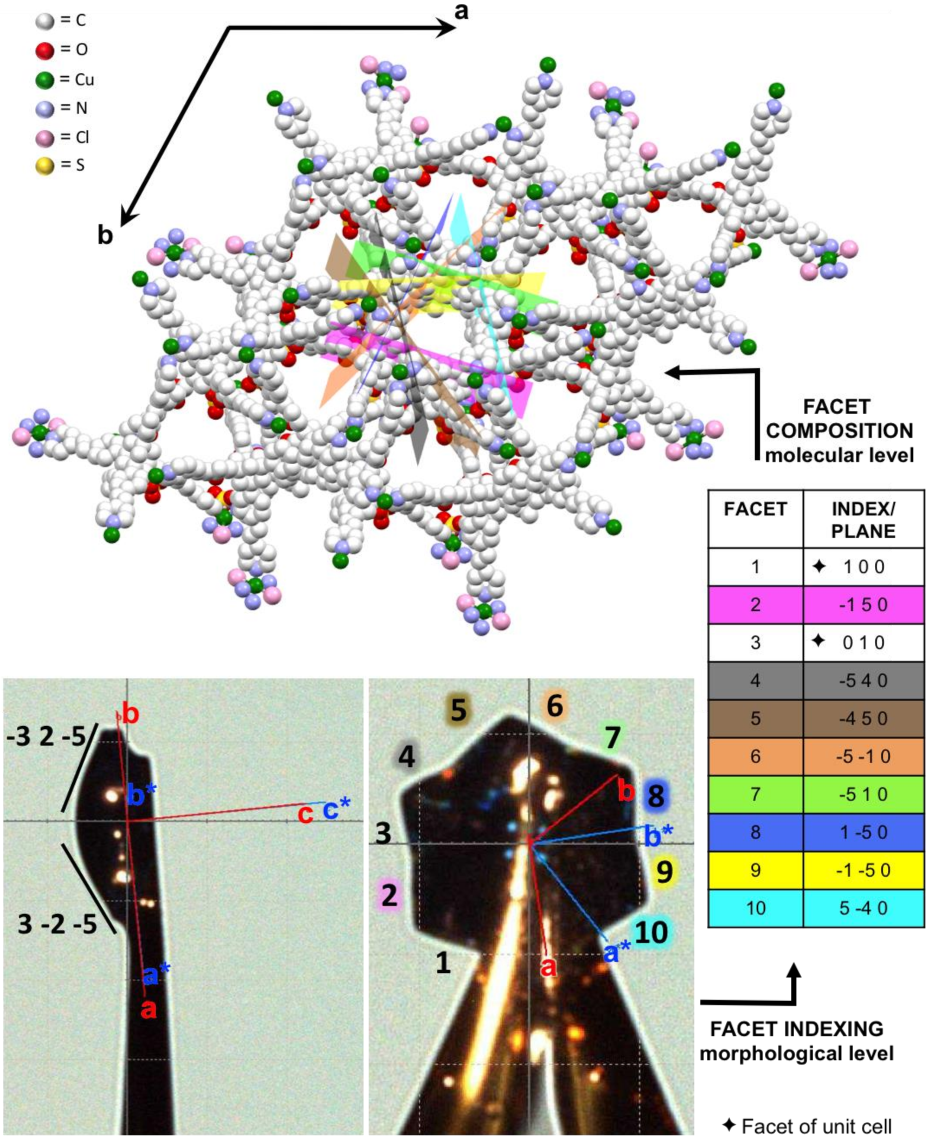

FACET	INDEX/ PLANE
1	✦ 1 0 0
2	-1 5 0
3	✦ 0 1 0
4	-5 4 0
5	-4 5 0
6	-5 -1 0
7	-5 1 0
8	1 -5 0
9	-1 -5 0
10	5 -4 0

Figure S12. Crystallographic structure down the *c* axis of **CSTAR-SO_4** (CIF v412, CCDC 2117031) (top). Optical microscopy images related to the side and top view of the same **CSTAR-SO_4** crystal mounted on a MiTeGen loop (bottom). Based on the crystal structure, the facet indexes and the orientation of the axes vs. the morphology of the mounted crystal were determined by CrysAlisPro 1.171.40.57a (Rigaku OD, 2019). The axes in the real (red) and reciprocal spaces (blue) are reported in the optical images. The top facets are marked in black and indexed in the optical image on the left. The lateral facets are labelled by numbers in the optical image on the right, and the relative *hkl* values are reported in the table.

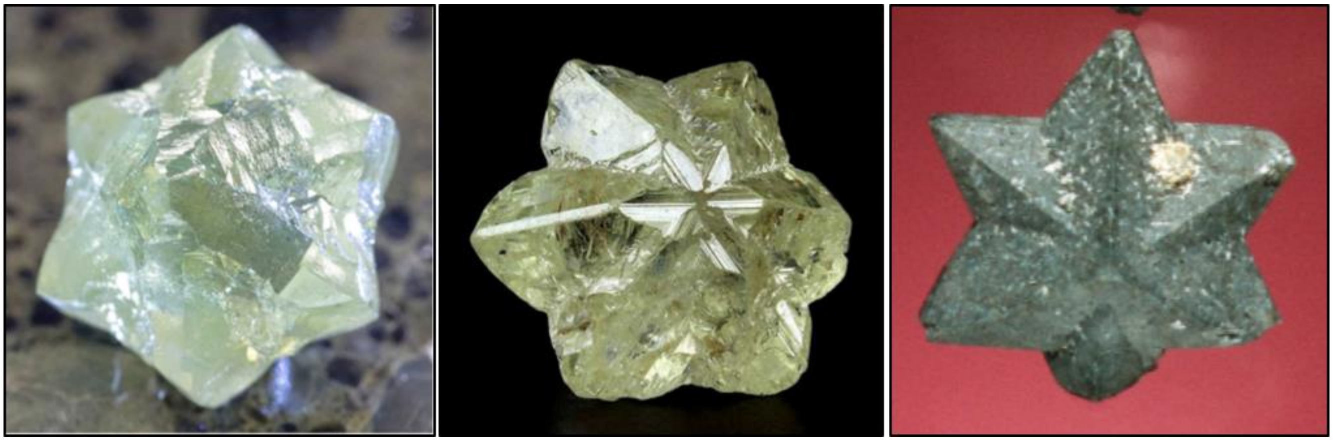

Figure S13. Examples of minerals with a star morphology and concave angles. Diamond (left), chrysoberyl (center) and benitoite (right).[S19-S21]

CSTAR-SO_4

EA wt%	C=72.48, H=4.13, N=5.71, Cu=5.53, Cl=3.58, S=0.1, O=8.47
EA formula	$C_{67}H_{45.6}N_{4.5}CuCl_{1.1}O_{5.9}S_{0.03}$

CSTAR-NO_3

EA wt%	C=71.68, H=4.45, N=5.55, Cu=6.92, Cl=2.23, O=9.17
EA formula	$C_{62}H_{42.3}N_{4.12}Cu_{1.1}Cl_{0.6}O_{5.9}$

Table S3. Elemental analysis data. Weight percentage of the elements constituting the bulk samples of **CSTAR-SO_4** and **CSTAR-NO_3** and their estimated formula. The presence of Cl^- can be attributed to the chloroform decomposition under the applied reaction conditions.[S4,S5,S22]

References

S1. Vasylyev, M.; Popovitz-Biro, R.; Shimon, L. J. W.; Neumann, R. Inorganic-organic hybrid materials based on Keggin type polyoxometalates and organic polyammonium cations. *J. Mol. Struct.* **2003**, *656*, 27–35.

S2. G. M. Sheldrick, A short history of SHELX. *Acta Crystallogr. Sect. A*. 2008, *64*, 112-122.

S3. A. L. Spek, PLATON SQUEEZE: a tool for the calculation of the disordered solvent contribution to the calculated structure factors. *Acta Crystallogr. Sect. C*. **2015**, *71*, 9–18.

S4. M. C. di Gregorio, L. J. W. Shimon, V. Brumfeld, L. Houben, M. Lahav, M. E. van der Boom, Emergence of chirality and structural complexity in single crystals at the molecular and morphological levels. *Nat Commun* **2020**, *11*, 380.

S5. Q. Wen, S. Tenenholtz, L. J. W. Shimon, O. Bar-Elli, L. Beck, L. Houben, S. R. Cohen, Y. Feldman, D. Oron, M. Lahav, M. van der Boom, Chiral and SHG-active metal-organic frameworks in solution and on surfaces: Uniformity, morphology control, oriented growth and post-assembly functionalization, *J. Am. Chem. Soc.* **2020**, *142*, 14210-14221.

S6. V. Singh, L. Houben, L. J. W. Shimon, S. R. Cohen, O. Golani, Y. Feldman, M. Lahav, M. E. van der Boom, Unusual surface texture, dimensions and morphology variations of chiral and single crystals. *Angew. Chem. Int. Ed.* **2021**, *60*, 18256–18264.

S7. Nasi, H.; Chiara di Gregorio, M.; Wen, Q.; Shimon, L. J. W.; Kaplan-Ashiri, I.; Bendikov, T.; Leitus, G.; Kazes, M.; Oron, D.; Lahav, M.; van der Boom, M. E. Directing the Morphology, Packing, and Properties of Chiral Metal–Organic Frameworks by Cation Exchange. *Angew. Chem. Int. Ed.* **2022**, *61*, e202205238.

S8. O. V Dolomanov, L. J. Bourhis, R. J. Gildea, J. A. K. Howard, H. Puschmann, OLEX2: a complete structure solution, refinement and analysis program. *J. Appl. Crystallogr.* **2009**, *42*, 339–341.

S9. D. Peck, A. Ostrander, Crystallography: The hexagonal system (2020), (available at https://www.mindat.org/article.php/2850/Crystallography%3A+The+Hexagonal+System)

S10. E. Koch, in *Prince E. (eds) International Tables for Crystallography Volume C: Mathematical, physical and chemical tables. International Tables for Crystallography, vol C* (Springer, Dordrecht, 2006).

S11. R. W. Cahn, Twinned crystals. *Adv. Phys.* **1954**, *3*, 363-445.

S12. W. H. Zachariasen, Multiple diffraction in imperfect crystals. *Acta Cryst.* **18**, 705-710 (1965).

S13. T. O. Yeates, in *Macromolecular Crystallography Part A* (Academic Press, 1997), vol. 276 of *Methods in Enzymology*, pp. 344–358.

S14. M. Catti, G. Ferraris, Twinning by merohedry and X-ray crystal structure determination. *Acta Crystallogr. Sect. A*. **1976**, *32*, 163–165.

S15. R. Herbst-Irmer, Twinning in chemical crystallography – a practical guide. *Zeitschrift für Krist. - Cryst. Mater.* **2016**, *231*, 573–581.

S16. D. C. Rees, The influence of twinning by merohedry on intensity statistics. *Acta Crystallogr. Sect. A*. **1980**, *36*, 578–581.

S17. Thompson M. C., in *Protein Crystallography. Methods in Molecular Biology, vol 1607.*, W. A., D. Z., J. M., Eds. (Humana Press, New York, 2017), pp. 185–217.

S18. E. Stanley, The identification of twins from intensity statistics. *J. Appl. Crystallogr.* **1972**, *5*, 191–194.

S19. Available at https://www.dia-designs.com/rough-diamond-oddities-are-chronicled-on-alrosas-instagram-page/

S20. Available at https://en.wikipedia.org/wiki/Chrysoberyl#/media/File:Chrysoberyl-282796.jpg

S21. Photo taken by Jeff Scovil and available at https://www.le-comptoir-geologique.com/benitoite encyclopedia.html
S22. L. Zhu, J. W. Bozzelli, Kinetics and mechanism for the thermal chlorination of chloroform in the gas phase: Inclusion of HCl elimination from $CHCl_3$ *Int. J. Chem. Kinet.* **2003** *35*, 647-660.
